# Bioprospecting of South African Plants as a Unique Resource for Bioactive Endophytic Microbes

**DOI:** 10.3389/fphar.2018.00456

**Published:** 2018-05-17

**Authors:** Muna Ali Abdalla, Lyndy J. McGaw

**Affiliations:** Phytomedicine Programme, Department of Paraclinical Sciences, Faculty of Veterinary Science, University of Pretoria, Pretoria, South Africa

**Keywords:** South African medicinal plants, secondary metabolites, endophytes, ethnobotanical approach, biological activities

## Abstract

South Africa has a long history and strong belief in traditional herbal medicines. Using ethnobotanical knowledge as a lead, a large number of South African medicinal plants have been discovered to possess a wide spectrum of pharmacological properties. In this review, bioprospecting of endophytes is highlighted by following the advantages of the ethnomedicinal approach together with identifying unique medicinal plants where biological activity may be due to endophytes. This review focuses on the current status of South African medicinal plants to motivate the research community to harness the benefits of ethnobotanical knowledge to investigate the presence of endophytic microbes from the most potent South African medicinal plants. The potential chemical diversity and subsequent putative medicinal value of endophytes is deserving of further research. A timely and comprehensive review of literature on recently isolated endophytes and their metabolites was conducted. Worldwide literature from the last 2 years demonstrating the importance of ethnobotanical knowledge as a useful approach to discover endophytic microbes was documented. Information was obtained from scientific databases such as Pubmed, Scopus, Scirus, Google Scholar, Dictionary of Natural Products, Chemical Abstracts Services, official websites, and scientific databases on ethnomedicines. Primary sources such as books, reports, dissertations, and thesises were accessed where available. Recently published information on isolated endophytes with promising bioactivity and their bioactive natural products worldwide (2015-2017) was summarized. The potential value of South African medicinal plants as sources of endophytes is discussed. The insights provided through this study indicate that medicinal plants in South Africa are highly under-investigated sources of potentially useful endophytic microbes. New approaches may be used by medicinal plant scientists for further exploration of natural products from endophytic fungi and bacteria in southern Africa.

## Introduction

Malaria, respiratory infections, HIV/AIDS, diarrhoeal diseases, and tuberculosis are leading transmissible and infectious diseases worldwide. Infections are still major human killers in the world, responsible for the mortality of around 50 000 people every day (Ahmad and Beg, 2001). An alarming prospect is that three main causes of death reported in pre-antibiotic America were tuberculosis, pneumonia, and gastrointestinal infections, and such diseases accounted for 30% of all deaths (Center for Disease Control and Prevention, 2013). The urgent need for new lead compounds with new modes of action against Gram-positive and Gram-negative bacteria is increasing to combat challenges of resistance against currently used drugs (Fair and Tor, 2014). In this context, the search for bioactive secondary metabolites from different plants and microorganisms as well as discovering new approaches such as microbial biotransformation, microbial co-culture, genome mining, and other molecular tools could be powerful strategies to find novel antibiotic candidates.

South Africa possesses a rich diversity of medicinal plants as well as knowledge of their use. It has been reported that 24 000 taxa exist in the region (Germishuizen and Meyer, 2003). Approximately 3 000 species are used as medicines, with about 350 most commonly used in traditional South African herbal medicine (Van Wyk et al., 2009). The unique Cape Floristic region in South Africa demonstrates the greatest extra-tropical concentration of higher plant species in the world. In this area 8 600 species are available and 68% of them were reported to be endemic (Kuete, 2013). It is important to note that the variety of South Africa's climate and altitude is responsible for its diversified flora and fauna. The vegetation regions of South Africa can be divided into four major types; forest and belts of palm in the east, south, and southwest coasts, the temperate grasslands which are located at the eastern portion of the interior plateau, the desert, and semi-desert natural region (Karoo) of the western interior and the final important natural habitat is the bushveld (savanna) which covers the Kalahari and the northeast (Worldmark Encyclopedia of the Nations, 2007). Of the estimated 200 natural orders of plants in the world, over 140 are reported. A huge variety of unique plants is found in the country—overall 200 species of euphorbia in the Cape Province alone, and over 500 species of grass. Wild flowers are one of the natural wonders of the world (including South Africa's national flower, the protea) and these grow in large quantities in the Cape region (Worldmark Encyclopedia of the Nations, 2007).

## Endophytes are potential sources of secondary metabolites

An endophyte is a bacterial or fungal species which lives within a plant and has an endosymbiotic relationship with it. All higher plants host a diversity of microbes and they are of growing interest as promising sources of biologically active agents. As a result, some of the metabolites isolated from plant sources trace their origin back to endophytic microbes within the plants, as plants and microorganisms may form intimate associations.

Endophytes can develop within roots, stems, or leaves and consequently colonize the internal plant tissues under the epidermal cell layers without causing any destructive diseases to their host (Strobel, 2003; Stone et al., 2004; Rodriguez et al., 2009; Jumpathong et al., 2010). Plants benefit from endophytic microbes as they synthesize natural products which promote plant growth and stimulate resistance against human pathogens (Bandara et al., 2006). Endophytes enhance plant growth in different ways by production of phytohormones, helping their host plants to tolerate environmental stresses and protecting from pathogens, pests, and herbivores by excreting alkaloids, which are toxic to insects and vertebrates (Schardl, 2001).

Although endophytic microbes have been comprehensively studied, the interaction between different endophytes and their hosts is poorly understood. A combination of factors, which could be environmental, genetic, and phenotypic, create diversiform endophytic microbiomes, but the benefits of these inner microbial communities to their host plants has been severely under-investigated (Compant et al., 2016). More hypothesis-driven studies will provide answers on how these close interactions between plants and endophytes do not cause disease. Investigation of endophytic microbes from medicinal plants is strategic because plants and microorganisms may form close associations and overlap may occur between metabolites from microbes and plants. Bioactive metabolites discovered from endophytes may be related to the independent development of these microorganisms. Alternatively, endophytes may have combined genetic information from their host plants, which stimulates their adaptability and enhances their defense mechanisms against pathogens and insects (Strobel, 2003; Pimentel et al., 2011).

Several antibiotics produced by endophytic actinobacteria isolated from medicinal plants have demonstrated a wide range of bioactivities against bacteria, fungi, and viruses. Additionally, these antibiotics exhibited activity at significantly low concentrations. These findings underline the promising and broad spectrum microbicidal potential of the secondary metabolites obtained from endophytic actinobacteria, mainly of the genus *Streptomyces* (Golinska et al., 2015). Moreover, numerous species of endophytic fungi have afforded a number of anticancer, antimicrobial, antidiabetic, insecticidal, and immunosuppressive compounds (Schneider et al., 2008). Interesting cyclotetrapeptides were isolated recently from endophytic fungi and bacteria (Abdalla, 2016, 2017).

The strong ethnomedicinal culture in South Africa, the great diversity of its plant species, their varying environments and unique biology and bioactivities serve as a wonderful unexplored source of potentially novel endophytic fungi and bacteria with diverse bioactivities and promising drug candidates. In this report we briefly discuss bioprospecting of South African medicinal plants exhibiting different biological activities to shed more light on the hidden area of endophytes from South African medicinal plants. The study focuses on the importance of ethnobotanical knowledge as an approach to discover endophytic microbes and describes some examples of current screening studies for endophytes and their secondary metabolites.

## Examples of previously discovered important plant and endophyte derived metabolites and their medicinal significance

**Figure 1:**
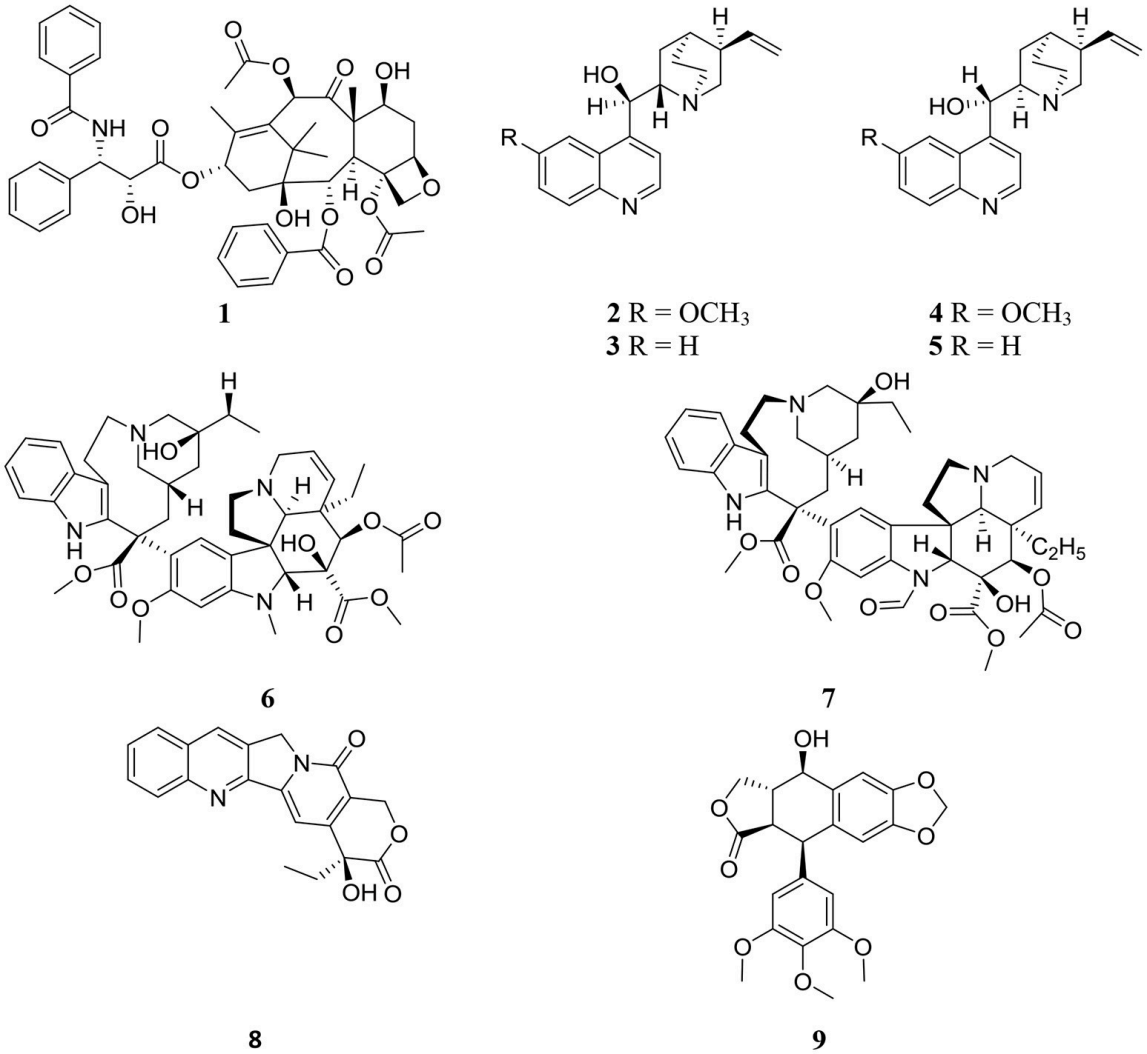
Structures of previously discovered plant and endophyte derived metabolites.

Structures of some important endophyte derived compounds are shown in Figure 1 and their sources and mode of action are summarized in Table 1. The discovery of the famous anticancer drug paclitaxel (taxol) (**1**), which is known to be produced by the yew tree *Taxus brevifolia*, from the endophytic fungus colonizing the related *Taxus wallichiana*, namely *Pestalotiopsis microspora*, focused attention on the importance of endophytes. Taxol is certainly the most facinating anticancer therapy for treatment of widespread types of human tumors such as breast, ovarian, prostate, head, neck, and lung cancers (Mekhail and Markman, 2002). Scientists have struggled for a long time to answer a real question: why do endophytic fungi harbored in yew trees make taxol? (Guo et al., 2008). A recent study provided the logical and plausible explanation that an endophytic fungus within the host plant responds to the entering pathogens and deploys specifically to the infection sites in the same way as the circulating immune cells in animals, secreting the antimicrobial compound (Soliman et al., 2015). Thus, endophytes protect their natural habitat within the host plant by secreting taxol in the extracellular spaces in hydrophobic bodies, as a very toxic compound to invading fungi. Another study confirmed the idea that all taxol-producing endophytic fungi have natural resistance to taxol, which possesses a potent antifungal activity against a plethora of fungal pathogens. This can explain the production of taxol by endophytic fungi as a fungicide to keep host plants healthy in a natural habitat (Kusari et al., 2014).

**Table 1:** Important drugs isolated from both endophytic fungi and their host plants.

**Name of the important drugs**	**Plant sources**	**Endophytic microbe sources**	**Trends in drug discovery**	**Mode of action**
Paclitaxel (taxol) (**1**)	1. Yew trees *Taxus brevifolia, Taxus* species and four genera of the family Taxaceae such as *Amenotaxus, Autrotaxus, Pseudotaxus*, and *Torreya*. 2.*Cephalotaxus* (Cephalotaxaceae). 3. *Podocarpus gracilor* Pilger (Podocarpaceae). 4. *Corylus avellena* L. (Betulaceae)	1.*Taxomyces andreanae* 2. More than 200 endophytic fungi producing Taxol harbored in *Taxus* non-*Taxus* species have been reported (Flores-Bustamante et al., 2010; Hao et al., 2013).	1. Taxol has been approved by Food and drug administration (FDA) for the advanced treatment of different human tumors, AIDS-related Kaposi's sarcoma. 2. Taxol (Registered trademark of Bristol Myers Squibb) is arguably the most used anti-tumor drug, which treats a number of carcinomas including breastcancer, ovarian, and pancreatic cancers (Talbot, 2015). 3. Discovered to be an antifungal and antioomycete drug, able to inhibit a wide range of serious pathogens (Talbot, 2015).	Prevents the breakdown of microtubules by interfering with the cell cycle. It is a potent drug against rapidly dividing tumor cells, reducing their growth and spread.
Quinine (**2**)	The bark of several species of *Cinchona* Trees, ca. 40 species of the family Rubiaceae. Example is *Cinchona ledgeriana* Moens ex Trimen	*Diaporthe* sp. (Maehara et al., 2011).	1.Important antimalarial drug for treatment of severe malaria and multidrug-resistant malaria. 2.Antimalarial, antiarrhythmic, and muscle relaxant drug (Song, 2009). 3. One of the *Cinchona* alkaloids, which have interesting and unique properties such as effective drugs, chiral catalysts, and selectors, indicators and bitter-taste compounds (Kacprzak, 2013).	*Cinchona* alkaloids act by interfering with the ability of *Plasmodium falciparum* for the digestion of hemoglobin during malaria life cycle. Generally vinca alkaloids are known to have cell cycle phase-specific (M and S phases) activity.
Cinchonidine (**3**)	The bark of several species of *Cinchona* trees, ca. 40 species of the family Rubiaceae. Example is *Cinchona ledgeriana* Moens ex Trimen	*Diaporthe* sp. (Maehara et al., 2011).	1.Antimalarial activity. 2. One of the *Cinchona* alkaloids, which have interesting and unique properties such as effective drugs, chiral catalysts, and selectors, indicators and bitter-taste compounds (Kacprzak, 2013).	
Quinidine (**4**)	The bark of several species of *Cinchona* Trees, ca. 40 species of the family Rubiaceae. Example is *Cinchona ledgeriana* Moens ex Trimen	*Diaporthe* sp. (Maehara et al., 2011)	1.Antimalarial, antiarrhythmic and muscle relaxant drug (Song, 2009). 2.One of the *Cinchona* alkaloids, which have interesting and unique properties such as effective drugs, chiral catalysts, and selectors, indicators and bitter-taste compounds (Kacprzak, 2013).	
Cinchonine (**5**)	The bark of several species of *Cinchona* Trees, ca. 40 species of the family Rubiaceae. Example is *Cinchona ledgeriana* Moens ex Trimen	*Diaporthe* sp. (Maehara et al., 2011)	1.Antimalarial activity. 2. One of the *Cinchona* alkaloids, which have interesting and unique properties such as effective drugs, chiral catalysts, and selectors, indicators and bitter-taste compounds (Kacprzak, 2013).	
Vinblastine (**6**) Vincristine (**7**)	*Catharanthus roseus*	*Fusarium oxysporum*	1.Anticancer therapies against various human tumors. 2.Have some immunosuppressant effects.	They work by binding to both soluble tubulin and tubulin in the microtubular proteins. This leads to the inhibition of polymerization of the microtubules and stops their formation during mitosis and cell devision at metaphase.
Camptothecin (**8**)	*Camptotheca acuminate*	*Fusarium solani*	1. A potent cancer chemotherapeutic drug due to its remarkable bioactivity against leukemias and different solid tumors. 2. The discovery of bioactivity of camptothecin against DNA topoisomerase I (Topo I) has established great breakthroughs. Accordingly camptothecin was listed on the frontlines of anticancer lead development in the late 1980s (Hsiang et al., 1985). 3. Synthesis of analogs and several studies of their pharmacology, formulation, preclinical, and clinical trials were greatly investigated (Ying-Qian et al., 2015).	Camptothecin works by inhibiting topoisomerase I (Topo I). This causes blocking at the rejoining step of the cleavage and religation reaction of Topo-I. This will lead to the accumulation of a covalent reaction intermediate, the cleavable complex (Liu et al., 2006)
Podophyllotoxin (**9**)	*Podophyllum* (*Sinopodophyllum*) *peltatum*	*Phialocephala fortinii*	Podophyllotoxin (**9**) is a precursor to the anticancer agents etoposide, teniposide, and etoposide phosphate (Canel et al., 2000). Podophyllotoxin (**9**) structure was modified later to inhance its solubility and inhibit its gastric toxicity; thus semisynthetic derivatives were discovered such as etoposide and teniposide (Eyberger et al., 2006).	Since the 1990s this compound was found to exhibit antineoplastic activity by reduction of tubulin polymerization and inhibition of mitosis during metaphase (Damayanthi and Lown, 1998).

The discovery of the famous *Cinchona* alkaloids, quinine (**2**), cinchonidine (**3**), quinidine (**4**), and cinchonine (**5**) from the endophytic filamentous fungus *Diaporthe* sp. harbored in the medicinal plant *Cinchona ledgeriana* has gained great interest. The findings demonstrated that *Cinchona* alkaloids are not only biosynthesized in *Cinchona* plant cells, but also in endophyte cells (Maehara et al., 2011). *Cinchona* alkaloids played an important role in human society as antimalarial drugs for more than 400 years (Song, 2009). Quinidine and cinchonine were the most active antimalarial drugs followed by quinine and cinchonidine. Although quinidine has been reported to exhibit 2 to 3-fold the antimalarial potency of quinine in both chloroquine sensitive and chloroquine-resistant strains of *Plasmodium falciparum* (Warhurst et al., 2003), it is not recommended for use as antimalarial therapy because of its cardiac activity. Accordingly quinine is approved as the most effective antimalarial drug.

The anticancer drugs vinblastine (**6**) and vincristine (**7**) have been obtained from the endophytic fungus *Fusarium oxysporum* which resides in a symbiotic relationship inside the medicinal plant *Catharanthus roseus* (Kumar et al., 2013). Vinblastine (**6**) and vincristine (**7**) are important components of vinca alkaloids in addition to vinorelbine and vindesine (Moudi et al., 2013). Vinca alkaloids are classified as the second most important class of anticancer agents and are used as original cancer therapies. The potent antineoplastic drug camptothecin (**8**) which is known to be produced by the medicinal plant *Camptotheca acuminata*, was also isolated from the endophytic fungus *Fusarium solani* obtained from the same plant (Kusari et al., 2009). Numerous previous publications have reported the pharmacological potential of camptothecin (**8**), which has therapeutic properties against AIDS and malaria in addition to uterine, colon, cervical, and ovarian cancer (Li et al., 2006).

Podophyllotoxin (**9**), a precursor of clinically important anticancer drugs, is not only obtained from the medicinal plant *Podophyllum* (*Sinopodophyllum*) *peltatum* but has also been discovered from the endophytic fungus *Phialocephala fortinii* isolated from the same plant (Eyberger et al., 2006). The isolation of podophyllotoxin (**9**) from the endophytic fungal culture can be optimized to obtain good quantities of the compound for further studies in drug development and clinical trials. The discovery of an endophytic fungus as a source of compound **9** will reduce the need to harvest the source plants.

From the above mentioned examples it can be assumed that endophytic fungi that are symbiotically harbored in host plants may have the capability to biosynthesize exactly the same secondary metabolites as their host plants. This could be due to the intergeneric genetic exchange that can be possible between the host plants and their endophytic fungi. As a result of continuous co-evolution and a symbiotic relationship with their host plants, it can be assumed that the endophytic fungi were adapted and familiarized to their special habitat. This may have led to gradual genetic variation, which allowed the endophytic fungi to take up some plant DNA segments into their own genomes. Morover endophytes can insert their own DNA segments into the host plant genomes. This can be a logical explanation of why endophytes have the ability to biosynthesize some secondary metabolites originating from their host plants (Gunatilaka, 2006; Zhang et al., 2006). The typical and historical example is the production of gibberellins from both fungi and plants (Choi et al., 2005).

## What makes South African medicinal plants a unique source to explore their endophytes?

### Interesting commericialized South African plants in the global markets

The indigenous medicinal plants of South Africa have been widely documented and investigated for their unique biology and chemical diversity. The Flora Capensis (a colonial flora) of South Africa was reported by Harvey and Sonder (1860,1862,1864) including important and ethnomedicinal uses of several species. Moreover, the exceptionally diverse and interesting South African flora has been known in the global market as the origin of many innovative products. Fifteen of the most important South African species were extensively discussed for their special commercial interest in the global market (Van Wyk, 2011). These plants are *Xysmalobium undulatum, Agathosma betulina, Aloe ferox, Aspalathus linearis, Artemisia afra, Cyclopia genistoide*s, *Hoodia gordonii, Harpagophytum procumbens, Hypoxis hemerocallidea, Lippia javanica, Mesembryanthemum tortuosum, Warburgia salutaris, Pelargonium sidoides, Siphonochilus aethiopicus*, and *Sutherlandia frutescens* (Van Wyk, 2011). These plants were produced as standardized products and are available in the international markets as tablets, capsules, teas, and tinctures (Van Wyk, 2011). Table 2 summarizes some commercially interesting South African plants and their importance.

**Table 2:** Some commercially interesting South African plants in the global market.

**Plant name**	**Family**	**Common name**	**Pharmacological properties**	**Chemical constituents**	**Commercialized product**
*Hoodia gordonii* (Masson) Sweet ex Decne	Apocynaceae	hoodia, ghaap	Has appetite-suppressant effect and used for weight management.	Glycosides of hoodigogenin A, calogenin glycosides, hoodistanal, dehydrohoodistanal, sterols, fatty acids, alcohols, and volatile compounds (Roza et al., 2013)	*Hoodia gordonii* slimming tablets 60 × 400 mg
*Mesembryanthemum tortuosum* L. (syn. *Sceletium tortuosum*)	Mesembryanthemaceae	kanna, kougoed	The plant has anti-anxiety and anti-depressant effects. In previous study the extract was found to be a strong blocker in 5-HT transporter binding assays (IC_50_ 4.3 μg/ml). It has also and had potent inhibitory effects on phosphodiesterase 4 (PDE4) (IC_50_ 8.5 μg/ml). Mesembrine has potent activity against the 5-HT transporter (K*_*i*_* 1.4 nM), while mesembrenone possessed activity against the 5-HT transporter and PDE4 (IC_50_'s < 1 μM). (Harvey et al., 2011)	Mesembrine, mesembrenone, mesembrenol, and tortuosamine	*Sceletium tortuosum* a brown powder used to relieve pain, stress and tension, and suppress anxiety
*Harpagophytum procumbens* (Burch.) DC. ex Meisn.	Pedaliaceae	devil's claw	1.Is a medicine for appetite Stimulation, dyspepsia, and degenerative disorders of the musculoskeletal system. 2. Clinical report has delivered its effectiveness for arthritis and rheumatic disorders. 3.A medicine used for liver and kidney disorders, as an oxytocic, purgative. In addition to other uses such as topical agent to treat wounds and skin rashes.	Iridoid glycosides (harpagoside, harpagide, and procumbide), sugars (tetrasaccharide, stachyose), triterpenoids (oleanolic and ursolic acid), phytosterols (beta-sitosterol), aromatic acids (caffeic, cinnamic, and chlorogenic acids), and flavonoids such as luteolin and kaempferol (Bradley, 1992).	Devil's Claw herbal supplements
*Agathosma betulina* (P.J.Bergius) Pillans	Rutaceae	Buchu	1.Has anti-inflammatory Activity. 2.The volatile oil of this plant exhibited potent activity against many diseases such as diabetes and nervousness. 3. It was exported to Britain in 1790. Later in 1821, it was listed in the British Pharmacopoeia as an important medicine for nephritis, urethritis, cystitis, and catarrh of the bladder.	Menthone, isomenthone, α-pinene, β-pinene, p-cymene, linalool and terpinen-4-ol, pulegone, *cis*-8-mercapto-*p*-menthan-3-one, *o*-methoxyphenol, and eugenol (Moolla, 2015)	Medico Buchu (*Agathosma betulina*) 50 ml. Carminative product which helps to relieve gas and bloating.
*Aspalathus linearis* (Burm.f.) R.Dahlgren	Fabaceae	rooibos tea	Has many pharmacological properties such as antispasmodic, antioxidant, anti-aging and anti-eczema, antimutagenic, anticarcinogenic, anti-inflammatory, antiviral, and antiatherosclerotic activities. (Canda et al., 2014).	Aspalathin, chrysoeriol, orientin, vitexin, and rutin (Gilani et al., 2006). In addition to 50 components of volatile constituents (Kawakami et al., 1993)	rooibos herbal tea and rooibos-derived commercial supplement (DCRS)
*Aloe ferox* Miller	Xanthorrhoeaceae	Cape Aloe, bitter Aloe	Has antioxidant (Loots et al., 2007) and anti-cancer (Van Wyk et al., 2009) properties and used as medicine for arteriosclerosis, hypertension and stress (Amusan et al., 2002) and arthritis and rheumatism (Van Wyk et al., 2009).	Aloe-emodin, *p*-hydroxyacetophenone, pyrocatechol, 7-hydroxy-2,5-dimethyl-chromone, furoaloesone, 2-acetonyl-8-(2-furoylmethyl)-7-hydroxy-5 methylchromone. (Kametani et al., 2007).	Natural products for skin care, bath and body, hair care and baby care. Products for digestive ailments, general wellbeing, and laxatives.

### Biological importance of South African plants

The efficacy and potency of South African medicinal plants has been studied in terms of both human and animal health. Extensive research has been conducted in different fields of interest in South Africa such as ethnomedicine, ethnoveterinary medicine, and phytomedicine to screen a wide variety of South African indigenous plants. Thus, very interesting biological activities of these plants have been discovered, such as antimicrobial efficacy, against a diverse range of Gram-positive and Gram-negative bacteria (Van Vuuren, 2008; Nyila et al., 2012; Okem et al., 2012; Mabona et al., 2013; Sharma and Lall, 2014; Aro et al., 2015; Elisha et al., 2016; Kabongo-Kayoka et al., 2016; Ramadwa et al., 2017) in addition to a large spectrum of fungi (Adamu et al., 2012; Otang et al., 2012). Moreover, anticancer (Sharma and Lall, 2014; Saeed et al., 2016), antioxidant (Saeed et al., 2012; Sharma and Lall, 2014; Dzoyem and Eloff, 2015), anti-inflammatory (Dzoyem and Eloff, 2015), anthelmintic (Aremu et al., 2010; Maphosa et al., 2010; Maphosa and Masika, 2012; Okem et al., 2012), antimalarial (Clarkson et al., 2004), antiviral (Bagla et al., 2012; Mehrbod et al., 2018), antidiabetic (Oyedemi et al., 2009; Kibiti, 2016), anti-HIV (Kamng'ona et al., 2011; Hurinanthan, 2013; Mthethwa et al., 2014) activities have been reported. Comprehensive reviews and surveys have discussed the potential of South African plants to treat inflammatory conditions (Iwalewa et al., 2007), diarrhea (Lin et al., 2002; Mathabe et al., 2006; Fawole et al., 2009; Bisi-Johnson et al., 2010; Semenya and Maroyi, 2012; Van Vuuren et al., 2015; Motlhatlego et al., 2018) and TB-related symptoms (McGaw et al., 2008).

In summary the unique diversity of South African plant species, in addition to their promising and potential bioactivities, is emphasized to alert researchers to the need to explore these plants as viable sources of endophytes for the production of potential drug candidates.

## Isolation and identification of endophytes

Acquiring endophytic fungi and bacteria with broad bioactivities requires selection criteria of the plant species. They should have ethnomedicinal history, unique biology, endemism, and special habitats (Yu et al., 2010). It is important to note that the plant should look apparently healthy, and young plant tissue is better than older material, especially in the case of isolation of endophytic fungi where slow-growing fungi are difficult to isolate (Strobel, 2003; Strobel and Daisy, 2003). Endophytes can be obtained from different plant parts such as seeds, leaves, and stems. Surface sterilization is a critical prerequisite in the most commonly used method for isolation of endophytes. This step is very important to eliminate possible contaminants, foreign material and fungal epiphytes and molds from plant tissues and encourage the growth of the internal microorganisms. Cutting plant material into many small pieces will increase the efficiency of sterilization and the isolation. Sterilization steps will be followed by the isolation of the endophytic fungi and bacteria on specific synthetic growth medium. Surface sterilization is a procedure in which plant material is treated with a surfactant such as ethanol (70–95%) followed by a strong surface sterilant for a short period; the household chlorine bleach (NaOCl) is usually used in concentrations of 2–10% after being diluted in water. The plant material is washed again with ethanol followed by several rinses in sterile water to remove residual sterilant. Ultrasonic cleaning equipment is an efficient surface sterilization protocol. A wide range of routine bacterial and mycological media are used for growth of bacteria and fungi, as well as isolation and subculturing for identification (Stone et al., 2004; Gagne-Bourque, 2011). For bacterial endophytes many media have been reported, such as Nutrient Agar (NA), Viande-Levure (Pavlo et al., 2011), or Rice extract Modified Rennie (RMR). For endophytic fungi malt extract agar medium is commonly used, alone or in combination with yeast extract (Stone et al., 2004).

**Figure 2:**
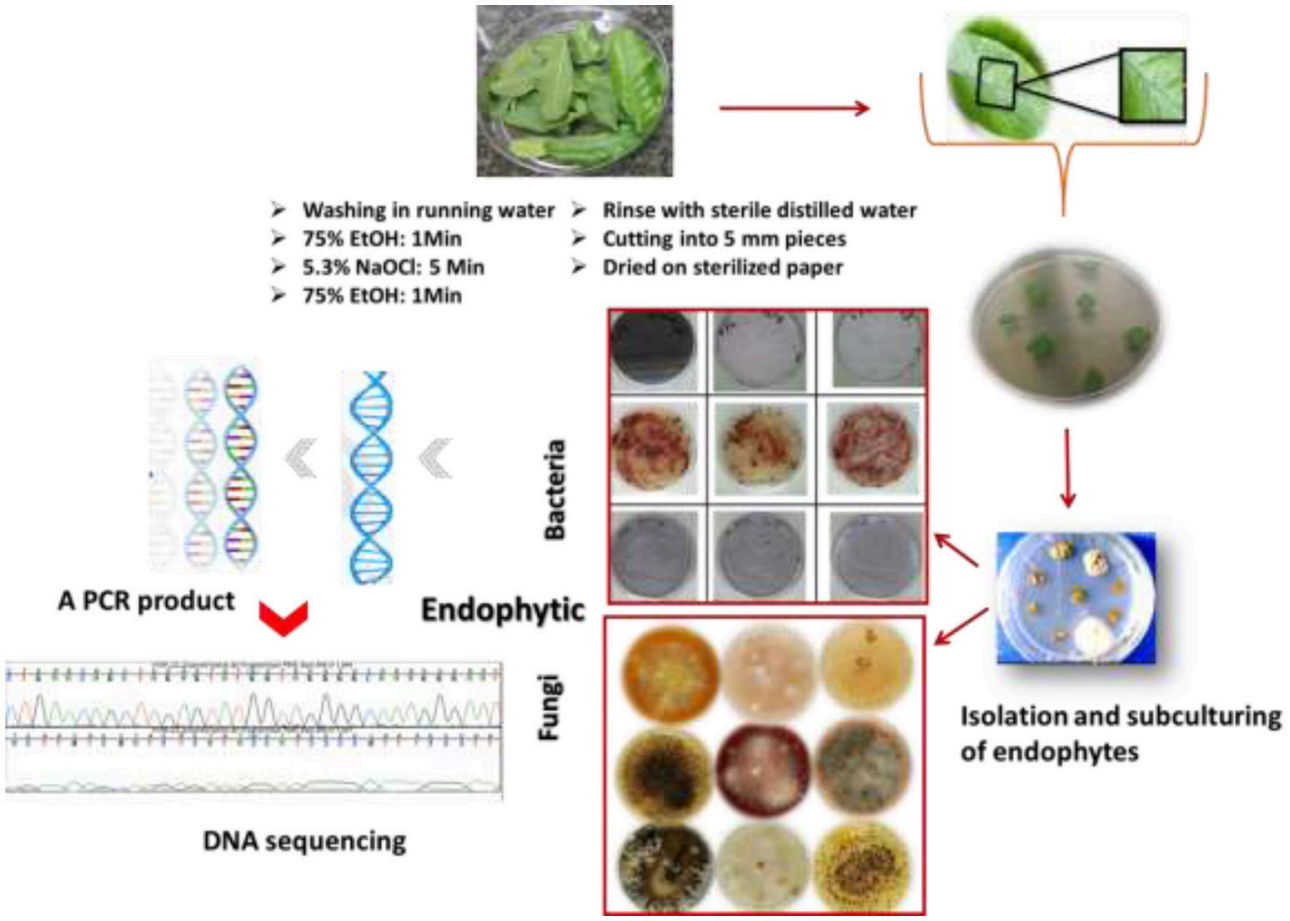
Overview of the procedure of isolation and identification of endophytic strains.

Optimal incubation conditions for fungi and bacteria are required to facilitate their primary growth and further sub-culturing steps. Based on their morphological and staining properties the successfully isolated endophytes are maintained as pure strains by several steps of sub-culturing processes. The purified bacteria and fungi are identified by means of morphological characteristics such as conidiogenesis and spore morphology in the case of fungi and texture opacity, color, form, and Gram-staining in the case of bacteria. Biochemical and molecular characterizations are additional techniques for endophyte identification. PCR amplification which requires universal primers targeting different conserved sequences of the rRNA genes and then DNA sequencing of the internal transcribed spacer (ITS) region is the most commonly used method for identification of a broad range of fungi to the species level (Schoch et al., 2012). For bacterial strains the universal method involves the PCR product of the 16S rRNA genes after designing suitable primers. A PCR product is sequenced and aligned and homology comparison is carried out using Genbank and other databases for identification. The procedure of isolation and characterization of endophytes is described in Figure 2.

## Why it is so challenging to cultivate endophytic microbes?

Micoorganisms such as epiphytes can be found on the plant surface, and also within plant tissues as endophytes. Numerous endophytic fungi and bacteria have shown beneficial effects on host plants, and this can be utilized in biotechnological applications. Additional research indicates that only 0.001–1% of endophytic bacteria are cultivable. Many isolated endophytic bacteria have reduced growth capacity (Alain and Querellou, 2009), are unculturable and need culture-independent techniques for detection and characterization (Liaqat and Eltem, 2016). In general, the majority of microorganisms are “uncultured” under laboratory controlled conditions (Lewis et al., 2010).

Difficulties in culturing many isolated endophytes may be attributed at least partly to residual plant metabolites, which are present during the isolation step but are not available during recultivation of the endophytes. It has been reported that crushed plant material contains molecules which are not present in the growth media, and such substances contribute to the growth of endophytic microbes (Eevers et al., 2015). Two types of endophytes can be found, namely obligate (Croes et al., 2013) and facultative endophytic microbes (Kamnev et al., 2005). Obligate endophytic strains are the hardest to grow due to their specific requirements. Developing new ways, approaches and mechanisms to cultivate unculturable endophytic and non-endophytic microorganisms is a top research priority in the field of microbial natural products. In this context it is very important to understand why unculturable microorganisms are uncultivated (Hurst, 2005). Strategies such as adopting single cell and high-throughput culturing procedures (Connon and Giovannoni, 2002; Nichols et al., 2008) better mimicking the natural milieu of the microrganisms (Bruns et al., 2002) and increasing the time of incubation and decreasing the concentration of nutrients have been used previously (Davis et al., 2005). The mixed fermentation or co-culture strategy has led to the discovery of the first class of growth factors for uncultured bacteria such as siderophores, and a previous study found that siderophores from neighboring microorganisms within the biofilm allowed uncultured bacteria to grow in a sand biofilm (Lewis et al., 2010). The technology of a diffusion chamber was used by NovoBiotic company (Cambridge, MA, USA) to induce the growth of uncultured actinomycetes (Lewis et al., 2010).

## Ethnobotanical knowledge is a scientific approach to discover endophytic microorganisms

The rich biodiversity of South African medicinal and indigenous plants together with the strong beliefs and practices of diverse ethnic groups (Van Wyk et al., 2009) have encouraged the South African research community to evaluate local indigenous plants for several biological activities such as antimicrobial, anticancer, anti-inflammatory, antioxidant, anti-HIV, and antimalarial properties. It is very important to mention that one of the most fascinating approaches used to discover promising endophytic strains is to follow ethnobotanical knowledge, where the ideas and experience of native people who usually rely on plants and plant materials as medicines and have a very long history must be considered (Abdalla and Matasyoh, 2014).

### Bioprospecting of chinese medicinal plants as a source of endophytes

The Chinese scientific research community recently began screening their medicinal plants with a focus on endophytic fungi and bacteria, their isolation, and bioactivities as new sources of natural lead compounds. Many examples have been reported, such as the endophytic streptomycetes, which were obtained from medicinal plants of the rainforest in Yunnan province, China, and were shown to have remarkable antitumour and antimicrobial activities (Li et al., 2008). One of the most interesting examples supporting the idea that endophytic microorganisms may be responsible for the medicinal properties of the particular plant species under study is the potent antimicrobial activity of the endophytic fungus *Fusarium proliferatum* which was isolated from the traditional Chinese medicinal plant *Celastrus angulatus* (Ji et al., 2005). In a screening study to investigate the potential of endophytes to produce bioactive secondary metabolites, the total DNA extract of endophytes isolated from 30 traditional Chinese herbs was used to screen PKS and NRPS gene clusters, and many of the endophytes under study were found to have the potential to produce bioactive secondary metabolites (Miller et al., 2012). The leaf of the Chinese endemic plant *Maytenus hookeri*, which is only found in the Yunnan area, afforded the endophytic fungus *Chaetomium globosum*, which delivered the anti-tuberculosis agent chaetoglobosin B (Ni et al., 2008).

### Discovery of the peptides munumbicins from endophytic streptomycetes in Australia

The ethnomedicinal approach afforded the peptides munumbicins, which were obtained from the endophytic *Streptomyces* NRRL 3052 isolated from the snakevine, *Kennedia nigriscans*. The latter plant was explored several years ago by a tribal leader, Reggie Munumbie, as a medicinal remedy in Aboriginal Australian culture to manage open, bleeding wounds to preclude sepsis. Currently, at least 39 different *Streptomyces* spp. have been discovered from several snakevine plants collected in different areas in the Northern Territory, Australia (Kunoh, 2002; Castillo et al., 2005). Moreover, most of the snakevine endophytic streptomycetes exhibit antimicrobial activity (Castillo et al., 2005). These findings support the premise that the world's rainforests are a great producer of endophytic streptomycetes.

### Plants of Latin America as a potential source of endophytes: Brazil and Mexico as examples

Around 20% or the estimated 300 000 plant species in the world exist in Brazil. Despite the diversity of Brazilian plants, studies on endophytic microbes from Brazil remain sparse. Several research groups are exploring endophytes from plants of not yet fully explored areas such as mangrove, Amazon, and Atlantic rain forests (Azevedo, 2014). It was reported that some endophytic fungi producing antimicrobial compounds were discovered from Brazilian mangroves (Sebastianes et al., 2012). Endophytes from Brazilian plants growing in the Amazon region and Atlantic forest have also been studied (Souza et al., 2004). A recent study explored ten actinomycetes from the medicinal plant *Vochysia divergens* found in the Pantanal sul-mato-grossense, an interesting unexplored area in Brazil. The Pantanal is well-known for its biological diversity in terms of its fauna and flora (Alho, 2008). This study discovered rare actinomycetes, which were never before reported as endophytes. The endophytic strains showed antimicrobial potential and were largely resistant to the antibiotics oxacillin and nalidixic acid (Gos et al., 2017). Among isolated strains the extract of the endophytic LGMB491 which is closely related to *Aeromicrobium ponti* exhibited remarkable bioactivity against methicillin-resistant *Staphylococcus aureus* (MRSA) with MIC of 0.04 mg/ml. Interestingly the strain delivered antibacterial compounds such as 1-acetyl-b-carboline, indole-3-carbaldehyde, 3-(hydroxyacetyl)-indole, brevianamide F and cyclo-(L-Pro-L-Phe) (Gos et al., 2017).

In Mexico the medicinal plant *Dendropanax arboreus* (L.) Decne. & Planch, which is known as Angelica tree and exsists in Mexico and South America, has ethnomedicinal uses in Mexico and Latin America such as managing fever, snakebites, and intestinal parasites (Bourdy et al., 2000). The anticancer compound falcarindiol, which showed potential activity against different types of cancer in animal models, was isolated from this plant (Setzer et al., 2000). Recently 45 endophytic fungi were obtained from *D. arboreus*. The crude extracts of the isolated endophytes such as *Corynespora, Endomelanconiopsis*, and *Thozetella* exhibited antifungal and antibacterial activity (Ramos-Garza et al., 2015). Moreover, a recent study in Mexico investigated the association of bacteria, *archaea* and fungi with the native cacti species *Myrtillocactus geometrizans* and *Opuntia robusta*, demonstrating the potential diversity of endophytes (Fonseca-García et al., 2016).

## Current screening studies for endophytes and their metabolites worldwide

**Figure 3:**
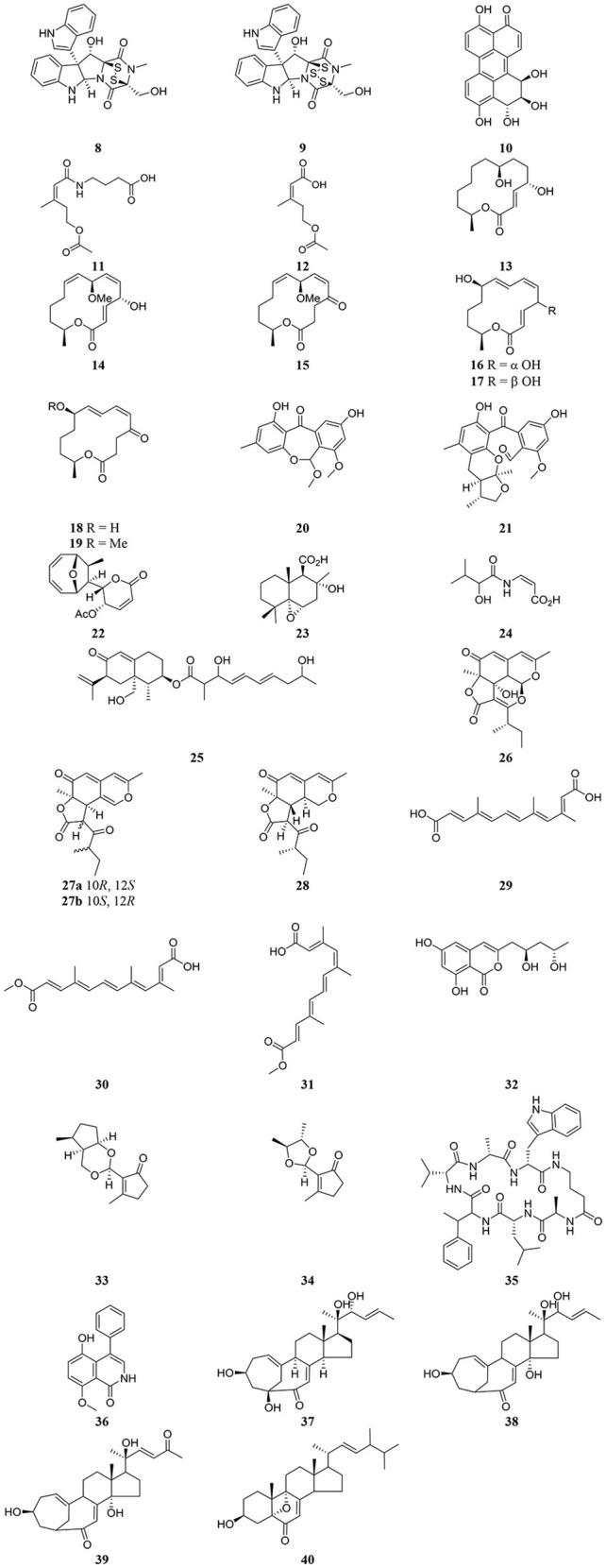
Structures of secondary metabolites isolated recently from endophytic microorganisms.

Table 3 provides a summary of global endophyte studies published in the last 2 years. *Glycyrrhiza glabra* L. is a famous medicinal plant used in traditional medicine worldwide for its ethnopharmacological characteristics to treat many ailments (Hosseinzadeh and Nassiri-Asl, 2015). In clinical studies and scientific research *G. glabra* and other species of the genus *Glycyrrhiza* exhibited potent pharmacological properties such as antimicrobial, anti-inflammatory, antioxidant, antiviral, antidiabetic, anti-asthma, and anticancer activities in addition to immunomodulatory, neuroprotective, hepatoprotective, gastroprotective, and cardioprotective applications (Hosseinzadeh and Nassiri-Asl, 2015). An endophytic fungus, *Phoma* sp. GG1F1 was isolated from *G. glabra* and its extract demonstrated significant antimicrobial activity. Two thiodiketopiperazines **8** and **9** (Figure 3) were obtained from the extract of the endophytic fungus *Phoma* sp. GG1F1 culture. Compounds **8** and **9** displayed very interesting bioactivities, as they decreased the growth of numerous bacterial pathogens such as *Staphylococcus aureus* and *Streptococcus pyogenes*, with IC_50_ values of less than 10 μM. Moreover, compounds **8** and **9** inhibited in a promising manner the biofilm formation in both pathogens. They afforded potent bactericidal activity in *in vitro* time kill kinetics. Compounds **8** and **9** are capable of acting synergistically with streptomycin and causing different effects in combination with ciprofloxacin and ampicillin. Additionally they are able to inhibit bacterial transcription/translation *in vitro*, and staphyloxanthin production in *S. aureus* (Arora et al., 2016).

**Table 3:** Current screening of endophytes, their isolated compounds, and bioactivities.

**Endophytic strain**	**Type**	**Plant name**	**Plant family**	**Isolated compounds**	**Ethnomedicinal uses of the plant**	**Bioactivity of the compounds**	**References**
1. *Phoma* sp. GG1F1	Fungus	*Glycyrrhiza glabra* L.	Fabaceae	Thiodiketopiperazines **8** and **9**	Famous worldwide for its ethnopharmacological properties to treat many ailments	Compounds inhibit the growth of *Staphylococcus aureus* and *Streptococcus pyogenes*, with IC_50_ values of less than 10 μM. Antibiofilm inhibition activities against several human pathogens. Both compounds acted synergistically with streptomycin and inhibited transcription/translation	Arora et al., 2016
2. *Sordariomycetes* sp. (PDA)BL3 3. *Sordariomycetes* sp. (PDA)BL5	Fungus	*Strobilanthes crispus* (L.) Bremek.	Acanthaceae	20 volatile metabolites identified from sample (PDA)BL3 and 21 volatile metabolites identify-ed from sample (PDA)BL5	Has importance in traditional medicine to manage diabetes, kidney stones, cancer and hypertension	Showed highest significant antimicrobial activity against 6 bacteria at 200 l g/disc whereas sample (PDA)BL5 has highest significant anticancer activity against all 5 cancer cell lines at concentrations ranging from 30 to 300 μg/ml	Jinfeng et al., 2017.
4. *Alternaria* species G7	Fungus	*Broussonetia papyrifera* (L.) Vent.	Moraceae	Altertoxin IV (**10**)	The fruits are edible. The leaves, fruit, and bark have a number of traditional medicinal uses	Compound **10** delivered weak antitumor activities	Zhang et al., 2016
5. *Aureobasidium pullulans*	Fungus	*Aloe vera* (L.) Burm.f.	Asphodelaceae	Pestalotiopamide E (**11**) and pestalotiopin B (**12**)	The plant is used in traditional herbal medicine for its regenerative, anti-microbial, anti-inflam-matory, and healing properties	Compounds **11** and **12** didn't show potential cytotoxic activity against mouse lymphoma L5178Y cells	El-Amrania et al., 2016
6. *Pestalotiopsis microspora*	Fungus	*Drepanocarpus lunatus* (L.f.) G.Mey.	Fabaceae	Pestalotioprolides C (**13**), D–H (**15**–**19**) and 7-*O*-methylnigrosporolide (**14**)	The plant is used for aphrodisiac, diarrhea, leprosy, purgative, and venereal diseases^a^.	Compounds **14**–**17** exhibited potent cytotoxicity against the murine lymphoma cell line L5178Y with IC_50_ values of 0.7, 5.6, 3.4, and 3.9 μM, respectively and compound **16** demonstrated highest activity against the human ovarian cancer cell line A2780 with an IC_50_ value of 1.2 μM	Liu et al., 2016
7. Xylaria sp.	Fungus	*Curcuma xanthorrhiza* Roxb. orth. var.	Zingiberaceae	Arugosin J (**20**) and xylarugosin (**21**)	The plant and the related *Curcuma longa* are used as spices and they are important medicinal plants. The well-known (Nuclear factor kappa B) NF-kB inhibitor curcumin was isolated from both species. It has strong anti-carcinogenic properties	Compounds **20** and **21** were in active (IC_50_ >50 μM) in the cytotoxicity test against the murine cancer cell line L5178Y by the MTT assay	Hammerschmidt et al., 2015
8. *Diaporthe* sp. SNB-GSS10	Fungus	*Sabicea cinerea* Aubl.	Rubiaceae	Mycoepoxydiene (**22**) altiloxin A (**23**), enamidin (**24**), and eremofortin F (**25**)	It is a medicinal plant of French Guiana and used as limb strengthener	Compound **22** demonstrated cytotoxic activity with IC_50_ values of 7.5, 17.7, and 15.8 μM against KB, MDA-MB-435, and MRC5 cancer cell lines, respectively. Compound **25** as well was cytotoxic on KB and MRC5 cells (IC_50_ = 13.9 and 12.2 μM. respectively). Compounds **23** and **24** were inactive (IC_50_ > 30 μM) on all tested cancer cell lines	Mandavid et al., 2015
9. *Colletotrichum* sp.BS4	Fungus	*Buxus sinica* (Rehder & E.H.Wilson) M.Cheng var. *insularis*	Buxaceae	Colletotrichones A–C (**26**–**28**)	The well-known Chine-se medicinal plant boxwood	Compound **26** showed remarkable antibacterial potencies against *Staphylococcus aureus* (DSM 799), *Escherichia coli* (DSM 1116), *Bacillus subtilis* (DSM 1088), and *Pseudomonas aeruginosa* (DSM 22644) with MIC of >10, 1.0, 0.1 and >10 μg/ml respectively comparable to the standard antibiotics streptomycin and gentamicin. Compound **28** displayed bioactivity against *E. coli* with MIC of >10 μg/ml. Compound **27a** demonstrated the same potency as streptomycin against the clinically relevant bacterium *S. aureus* (MIC = 5.0 μg/ml). None of the individual compounds showed *in vitro* cytotoxicity on a human acute monocytic leukemia cell line (THP-1)	Wang et al., 2016
10. *Nectria* sp. HN001	Fungus	*Sonneratia ovata* Backer	Lythraceae	Nectriacids A–C (**29**–**31**) and 12-epicitreoisocoumarinol (**32**)	The fermented juice is useful for hemorrhages and the fruit is applied in poultices to treat sprain Useful Tropical Plants Database, 2014	Compounds **30** and **31** showed remarkable α-glucosidase inhibitory activity in comparison to positive control (acarbose, IC_50_, 815.3 μM), with IC_50_ values of 23.5 and 42.3 μM, respectively	Cui et al., 2016
11. *Endomelanconiopsis endophytica*	Fungus	*Ficus hirta* Vahl	Moraceae	Endomeketals A–B (**33**–**34**)	The milky latex of the plant is used to treat wounds. While the decoction of the stem bark is applied for fevers. A paste of the roots and fruit is applied for wound of snakebites Useful Tropical Plants Database, 2014	Compounds **33** and **34** were in active against the following human cancer cell; SF-268, MCF-7, NCI-H460, and HepG-2	Sun et al., 2016
12. *Mucor irregularis*	Fungus	*Moringa stenopetala* (Baker f.) Cufod.	Moringaceae	Unguisin F (**35**)	The plant has multipurposes uses for the community. The seeds are used as purifiers of turbid water in Africa Mekonnen and Gessesse, 1998	Compound **35** didn't show significant antibacterial or antifungal activity	Akone et al., 2016
13. *Penicillium* sp. R22	Fungus	*Nerium indicum* Mill.	Apocynaceae	5-hydroxy-8-methoxy-4-phenylisoquinolin-1(2*H*)-one (**36**)	The medicinal importance of the plant dates back to 1500 years BC. It is used all over the world to treat dermatitis, eczema, psoriasis, herpes, sores, abscesses, warts, corns, skin cancer, ringworm, scabies, epilepsy, asthma, malaria, and heart disease Isaacs, 2008; Dey and Chaudhuri, 2014.	Compound **36** displayed weak antibacterial activity	Ma et al., 2017
14. *Chaetomium* sp. M453	Fungus	*Huperzia serrata* (Thunb.) Trevis.	Huperziaceae	Neocyclocitrinols E-G (**37-39**) and 3β-hydroxy-5,9-epoxy-(22E,24R)-ergosta-7,22-dien-6-one (**40**)	The plant is known in Chinese traditional medicine as an anti-inflammatory remedy for treating pain and swelling after trauma. It is also removing heat and exhibited detoxification effects. Huperzine A was obtained from *Huperzia serrata* and showed many pharmacological properties Ho et al., 2011	Compounds **37**-**40** were tested for cytotoxicity and acetylcholinesterase (AChE) inhibitory activities. None of the compounds displayed any cytotoxicity at 40 μM. Compound **35** exhibited weak AChE inhibitory activity	Yu et al., 2017

a *Dr. Duke's Phytochemical and Ethnobotantical Databases. National Agricultural Library. USA. https://phytochem.nal.usda.gov/phytochem/search*.

*Strobilanthes crispus* is a medicinal plant used in traditional medicine to treat diabetes, kidney stones, cancer, and hypertension and it has antimicrobial activities (Jinfeng et al., 2017). Two fungal endophytes closely related to *Sordariomycetes* sp. were obtained from *S. crispus*. Both endophytic fungi, named *Sordariomycetes* sp. (PDA)BL3 and *Sordariomycetes* sp. (PDA)BL5 exhibited potent antimicrobial activity against 6 bacterial strains at 200 μg/disc and high anticancer activity against 5 cancer cell lines at concentrations ranging from 30 to 300 μg/ml, respectively. Twenty volatile metabolites were obtained from the endophytic fungus *Sordariomycetes* sp. (PDA)BL3 and 21 volatile metabolites were noted from the second endophytic fungus *Sordariomycetes* sp. (PDA)BL5 using gas chromatography coupled with mass spectrometry (GC–MS). The study recommended that further identification of the compounds responsible for the interesting antimicrobial and anticancer activities of both endophytic fungi should be done.

Altertoxin IV (**10**) was recently discovered from the ethyl acetate extract of a culture of the endophytic fungus *Alternaria* species G7 in *Broussonetia papyrifera* along with other previously known cytotoxic compounds. Altertoxin IV (**10**), a new member of the perylenequinone derivatives, exhibited weak antitumor activities (Zhang et al., 2016). An amide pestalotiopamide E (**11**) and its corresponding acid pestalotiopin B (**12**) were isolated from the mycelial extract of the endophytic fungus *Aureobasidium pullulans* cultured from leaves of *Aloe vera* (Asphodelaceae) collected from Marrakesh (Morocco) (El-Amrania et al., 2016).

Recently, seven new 14-membered macrolides, pestalotioprolides C (**13**), D–H (**15**–**19**) and 7-*O*-methylnigrosporolide (**14**) were isolated from the mangrove-derived endophytic fungus *Pestalotiopsis microspora* obtained from fresh fruits of the mangrove plant *Drepanocarpus lunatus* (Fabaceae) collected in Cameroon (Liu et al., 2016). Compounds **14**–**17** showed significant cytotoxicity against the murine lymphoma cell line L5178Y with IC_50_ values of 0.7, 5.6, 3.4, and 3.9 μM respectively, while compound **16** showed potent activity against the human ovarian cancer cell line A2780 with an IC_50_ value of 1.2 μM (Liu et al., 2016).

The endophytic fungus *Xylaria* sp. isolated from the medicinal plant *Curcuma xanthorrhiza*, collected on the island of Timor, Indonesia afforded two new compounds, arugosin J (**20**) and xylarugosin (**21**). The two metabolites **20** and **21** were inactive (IC_50_ > 50 μM) when tested for cytotoxicity against the murine cancer cell line L5178Y by the MTT assay (Hammerschmidt et al., 2015). The *Diaporthe* genus is the sexual form of *Phomopsis*, and these are important endophytic fungi obtained from tropical and temperate woody plants. They have a potential role in protecting plants from fungal infection (Mandavid et al., 2015).

The endophytic fungus *Diaporthe* sp. SNB-GSS10 which was isolated from a medicinal plant *Sabicea cinerea* delivered four bioactive componds mycoepoxydiene (**22**) altiloxin A (**23**), enamidin (**24**), and eremofortin F (**25**) (Mandavid et al., 2015). Compound **22** displayed cytotoxic activity with IC_50_ values of 7.5, 17.7, and 15.8 μM against KB, MDA-MB-435, and MRC5 cancer cell lines, respectively. Compound **25** was also cytotoxic to KB and MRC5 cells (IC_50_ = 13.9 and 12.2 μM, respectively). Unfortunately compounds **23** and **24** were inactive (IC_50_ > 30 μM) on all tested cancer cell lines (Mandavid et al., 2015). Colletotrichones A–C (**26**–**28**) were isolated from the endophytic fungus *Colletotrichum* sp.BS4, obtained from the leaves of the famous Chinese medicinal plant boxwood, *Buxus sinica* (Wang et al., 2016). Compound **26** showed remarkable antibacterial potencies against environmental bacteria in comparison to the standard antibiotics streptomycin and gentamicin. Compound **28** displayed bioactivity against *E. coli*. Compound **27a** demonstrated the same potency as streptomycin against the clinically relevant bacterium *S. aureus*. None of the individual compounds showed *in vitro* cytotoxicity on a human acute monocytic leukemia cell line (THP-1) (Wang et al., 2016). Due to the discovery of the very effective compounds **26**-**28** which are related to the azaphilones group, the study suggested that more investigation should be done on the endophyte-mediated host chemical defense against pathogens. As the endophytic fungus was obtained from the leaves of *B. sinica*, the azaphilone compounds may provide an example of natural product-mediated chemical defense to the host plant species against different bacteria.

The endophytic fungus *Nectria* sp. HN001, which was obtained from the mangrove plant *Sonneratia ovata* collected from the South China Sea, delivered nectriacids A–C (**29**–**31**) and 12-epicitreoisocoumarinol (**32**), along with three known compounds. Compounds (**29**-**32**) were tested against α-glucosidase inhibitory activity by UV absorbance at 405 nm. Compounds **30** and **31** showed potent inhibitory activity (Cui et al., 2016). Endomeketals A–B (**33**–**34**) were isolated from the endophytic fungus *Endomelanconiopsis endophytica* A326 harbored in *Ficus hirta* (Sun et al., 2016). *E. endophytica* was discovered as a new anamorph genus in the Botryosphaeriaceae. Unfortunately compounds **33** and **34** didn't display cytotoxic activity against 4 tumor cell lines, SF-268, MCF-7, NCI-H460, and HepG-2. The endophytic fungus *Mucor irregularis* harbored in the medicinal plant *Moringa stenopetala*, collected in Cameroon, afforded the new cyclic heptapeptide unguisin F (**35**) (Akone et al., 2016). Compound **35** didn't show significant antimicrobial activity. The endophytic fungus *Penicillium* sp. R22 was isolated from the root of the medicinal plant *Nerium indicum* collected from Qinling Mountain, Shaanxi Province, China.

A new secondary metabolite named 5-hydroxy-8-methoxy-4-phenylisoquinolin-1(2*H*)-one (**36**) was discovered from *Penicillium* sp. R22 together with other known isoquinoline alkaloid derivatives. Compound **36** displayed weak antibacterial activity (Ma et al., 2017). Unusual C_25_ steroids named as neocyclocitrinols E-G (**37**-**39**), together with 3β-hydroxy-5,9-epoxy-(22E,24*R*)-ergosta-7,22-dien-6-one (**40**) were isolated from the endophytic fungus *Chaetomium* sp. M453 obtained from the Chinese herbal medicine *Huperzia serrate* (Yu et al., 2017). To our knowledge all natural products have an essential function in their respective habitat and random screening may be the only way to discover this activity. That means when a natural compound doesn't exhibit antimicrobial or anticancer activities it perhaps has other biological applications, which should be discovered.

In conclusion, endophytic fungi have been found to be a major source of phytochemicals and numerous bioactive secondary metabolites as also reported by Machavariani and Terekhova (2014). Owing to the discovery of the above-mentioned metabolites which were obtained from endophytic fungi, it can be assumed that endophytic fungi have very exciting possibilities to produce a plethora of novel bioactive natural products (Kusari et al., 2012). These findings highlight the importance of chemical communication strategies of endophytic fungi with their host plants and with other endophytic associations and their role in the activation of cryptic secondary metabolism pathways in endophytic fungi (Scherlach and Hertweck, 2009; Walsh and Fischbach, 2010). These lead to the varied biological diversity of endophytes, especially fungi, and their capability to produce bioactive molecules, which highlights a number of interesting research areas on endophytes.

## Future perspectives

Until now little has been achieved worldwide in the research area of endophytic fungi and bacteria, and few strains have been isolated, implying that the opportunity to find promising strains and their novel natural products in different niches and ecosystems is huge. Plants growing in areas of great biodiversity have the highest potential for housing endophytes.

South Africa, which represents less than 1% of the world's land surface, possesses 8% of its plant species. It attracts the scientific community due to the rich plant biodiversity, as it contains over 20 000 different species (Cherry, 2005). In the longer term, the unique diversity of South African plants serves as a great source for discovering the endophytic biodiversity, their secondary metabolites, and more about the interesting relationship between endophytes and their host plants. Since endophytes from South African medicinal plants have not been studied in any detail, there is a need to screen them for production of bioactive metabolites as they are often easily cultured and cultivated in the laboratory instead of collecting the plants and hence affecting the environment. Endophytes could also be responsible for the medicinal properties of the plants; in this regard interesting scientific scenarios will be discovered by studying their bioactivities as well as their secondary metabolites. This may lead to the isolation of new drug candidates, especially from those potential plants, as the endophytic compounds may be responsible for the entire activity.

## Author contributions

MA conceptualized the idea, wrote, and edited the manuscript and LM provided input during preparation, edited, and submitted the manuscript.

### Conflict of interest statement

The authors declare that the research was conducted in the absence of any commercial or financial relationships that could be construed as a potential conflict of interest.
